# CUDASW++: optimizing Smith-Waterman sequence database searches for CUDA-enabled graphics processing units

**DOI:** 10.1186/1756-0500-2-73

**Published:** 2009-05-06

**Authors:** Yongchao Liu, Douglas L Maskell, Bertil Schmidt

**Affiliations:** 1 School of Computer Engineering, Nanyang Technological University, Singapore

## Abstract

**Background:**

The Smith-Waterman algorithm is one of the most widely used tools for searching biological sequence databases due to its high sensitivity. Unfortunately, the Smith-Waterman algorithm is computationally demanding, which is further compounded by the exponential growth of sequence databases. The recent emergence of many-core architectures, and their associated programming interfaces, provides an opportunity to accelerate sequence database searches using commonly available and inexpensive hardware.

**Findings:**

Our CUDASW++ implementation (benchmarked on a single-GPU NVIDIA GeForce GTX 280 graphics card and a dual-GPU GeForce GTX 295 graphics card) provides a significant performance improvement compared to other publicly available implementations, such as SWPS3, CBESW, SW-CUDA, and NCBI-BLAST. CUDASW++ supports query sequences of length up to 59K and for query sequences ranging in length from 144 to 5,478 in Swiss-Prot release 56.6, the single-GPU version achieves an average performance of 9.509 GCUPS with a lowest performance of 9.039 GCUPS and a highest performance of 9.660 GCUPS, and the dual-GPU version achieves an average performance of 14.484 GCUPS with a lowest performance of 10.660 GCUPS and a highest performance of 16.087 GCUPS.

**Conclusion:**

CUDASW++ is publicly available open-source software. It provides a significant performance improvement for Smith-Waterman-based protein sequence database searches by fully exploiting the compute capability of commonly used CUDA-enabled low-cost GPUs.

## Background

In bioinformatics, sequence database searches are used to find the similarity between a query sequence and subject sequences in the database so as to identify evolutionary relationships. The sequence similarities can be determined by computing their optimal local alignments using the dynamic programming based Smith-Waterman (SW) algorithm [1,2]. However, the cost of this approach is expensive in terms of computing time and memory space. This is especially evident with the rapid growth of biological sequence databases demanding powerful high-performance computing solutions. Due to the computationally demanding nature of the SW algorithm, some heuristic solutions, such as FASTA [3] and BLAST [4,5], have been devised to improve the execution speed, but at the expense of sensitivity. This may result in some distantly related sequences not being detected.

The recent emergence of accelerator technologies and many-core architectures, such as FPGAs, Cell/BEs and GPUs, provides the opportunity to significantly reduce the runtime for many bioinformatics programs on commonly available and inexpensive hardware. Modern implementations of the SW algorithm are mainly focused on these new technologies, including FPGA [6-8], SSE2 [9,10], Cell/BE [10-12], GPU [13] and CUDA [14].

Oliver et al. [6,7] constructed a linear systolic array to perform the SW algorithm on a standard Virtex II FPGA board, achieving a peak performance of about 5 GCUPS using affine gap penalties. Li et al. [8] exploits custom instructions to accelerate the SW algorithm on an Altera Stratix EP1S40 FPGA by dividing the SW matrix into grids of 8 × 8 cells and achieved an estimated peak performance of about 23.8 GCUPS for DNA sequences. Farrar [9] exploited the SSE2 instruction set to compute the SW algorithm in a striped pattern, outperforming the previous SIMD based SW implementations by Wozniak [15] and Rognes [16]. This striped SW approach was then optimized for Cell/BE [11]. SWPS3 [10] extends Farrar's work for Cell/BE and also improves it for x86/SSE2 to support multi-core processors. CBESW [12] was designed for the Cell/BE-based PlayStation 3 (PS3) and achieves a peak performance of about 3.6 GCUPS.

The first implementation of the SW algorithm on GPUs was developed by Liu et al. [13] using OpenGL. However, the introduction of CUDA and CUDA-enabled GPUs has resulted in a simpler and more efficient methodology for performing scientific computing on GPUs. Therefore, SW-CUDA [14] has been implemented using two NVIDIA GeForce 8800 GTX graphics cards and achieves a peak performance of about 3.5 GCUPS.

In this paper, the compute power of CUDA-enabled GPUs is further explored to accelerate SW sequence database searches. Two versions of CUDASW++ are implemented: a single-GPU version and a multi-GPU version. The single-GPU version achieves a performance of close to 10 GCUPS on an NVIDIA GeForce GTX 280 (GTX 280) graphics card and the multi-GPU version achieves a performance of up to 16 GCUPS on an NVIDIA GeForce GTX 295 (GTX 295) graphics card. Our CUDASW++ implementations provide better performance guarantees for protein sequence database searches compared to SWPS3 [10], CBESW [12], SW-CUDA [14] and NCBI-BLAST (version 2.2.19) [5].

### The Smith-Waterman Algorithm

The SW algorithm is exploited to identify the optimal local alignment between two sequences by computing the similarity score by means of dynamic programming. Given two sequences *S*_*a *_and *S*_*b *_of lengths *l*_a _and *l*_b _respectively, the SW algorithm computes the similarity score *H*(*i*, *j*) of two sequences ending at position *i *and *j *of *S*_*a *_and *S*_*b*_, respectively. *H*(*i*, *j*) is computed as in equation (1), for 1 ≤ *i *≤ *l*_a_, 1 ≤ *j *≤ *l*_b_:

(1)

where, *sbt *is the character substitution cost table, *ρ *is the gap opening penalty and *σ *is the gap extension penalty. The recurrences are initialized as *H*(*i*, 0) = *H*(0, *j*) = *E*(*i*, 0) = *F*(0, *j*) = 0 for 0 ≤ *i ≤ l*_a _and 0 ≤ *j ≤ *= *l*_b_. The maximum local alignment score *maxScore *is defined as the maximum value in matrix *H*. The calculation of *H*(*i*, *j*) is shown graphically in Additional file 1, with the three arrows showing the data dependencies in the alignment matrix: each cell depends on its left, upper, and upper-left neighbors. This dependency implies that all cells on the same minor diagonal in the alignment matrix are independent from each other and can be computed in parallel (see Additional file 1). Thus, the alignment can be computed in minor-diagonal order from the top-left corner to the bottom-right corner in the alignment matrix. Note that, in order to calculate minor diagonal *i *only the results of the minor diagonal *i*-1 and *i*-2 are necessary and therefore *maxScore *can be found in linear space.

### The CUDA Programming Model

CUDA (Compute Unified Device Architecture) is an extension of C/C++ which enables users to write scalable multi-threaded programs for CUDA-enabled GPUs [17]. CUDA programs can be executed on GPUs with NVIDIA's Tesla unified computing architecture [18].

CUDA programs contain a sequential part, called a *kernel*. The kernel is written in conventional scalar C-code. It represents the operations to be performed by a single thread and is invoked as a set of concurrently executing threads. These threads are organized in a hierarchy consisting of so-called thread blocks and grids (see Additional file 2). A *thread block *is a set of concurrent threads and a *grid *is a set of independent thread blocks. Each thread has an associated unique ID (*threadIdx*, *blockIdx*) ∈ {0,..., *dimBlock*-1} × {0,..., *dimGrid*-1}. This pair indicates the ID within its thread block (*threadIdx*) and the ID of the thread block within the grid (*blockIdx*). Similar to MPI processes, CUDA provides each thread access to its unique ID through corresponding variables. The total size of a grid (*dimGrid*) and a thread block (*dimBlock*) is explicitly specified in the kernel function-call:

kernel<<<*dimGrid*, *dimBlock*, *other configurations*>>> (parameter list);

The hierarchical organization into blocks and grids has implications for thread communication and synchronization. Threads within a thread block can communicate through a *per-block shared memory *(PBSM) and may synchronize using barriers. However, threads located in different blocks cannot communicate or synchronize directly. Besides the PBSM, there are four other types of memory: per-thread private local memory, global memory for data shared by all threads, texture memory and constant memory. Texture memory and constant memory can be regarded as fast read-only caches.

The Tesla architecture supports CUDA applications using a scalable processor array. The array consists of a number of *streaming multiprocessors *(SMs). Each SM contains eight scalar processors (SPs), which share a PBSM of size 16 KB (see Additional file 3). All threads of a thread block are executed concurrently on a single SM. The SM executes threads in small groups of 32, called *warps*, in single-instruction multiple-thread (*SIMT*) fashion. Thus, parallel performance is generally penalized by data-dependent conditional branches and improves if all threads in a warp follow the same execution path.

In order to write efficient CUDA applications, it is important to understand the different types of memory spaces in more detail.

• *Readable and writable global memory *is relatively large (typically 1 GB), but has high latency, low bandwidth, and is not cached. The effective bandwidth of global memory depends heavily on the memory access pattern, e.g. coalesced access generally improves bandwidth.

• *Readable and writable per-thread local memory *is of limited size (16 KB per thread) and is not cached. Access to local memory is as expensive as access to global memory and is always coalesced.

• *Read-only constant memory *is of limited size (totally 64 KB) and cached. The reading cost scales with the number of different addresses read by all threads. Reading from constant memory can be as fast as reading from a register (e.g. if all threads of a half-warp read the same address).

• *Read-only texture memory *is large (depending on the size of global memory) and is cached. Texture memory can be read from kernels using texture fetching device functions. Reading from texture memory is generally (not absolutely) faster than reading from global or local memory.

• *Readable and writable per-block shared memory *is fast on-chip memory of limited size (16 KB per block). Shared memory can only be accessed by all threads in a thread block. Shared memory is divided into equally-sized banks that can be accessed simultaneously by each thread. Accessing the shared memory is as fast as accessing a register as long as there are no bank conflicts.

• *Readable and writable per-thread registers *are the fastest memory to access but is of very limited size.

## Findings

### Methods

Considering the optimal local alignment of a query sequence and a subject sequence as a task, we have investigated two approaches for parallelizing the sequence database searches using CUDA.

• *Inter-task parallelization*. Each task is assigned to exactly one thread and *dimBlock *tasks are performed in parallel by different threads in a thread block.

• *Intra-task parallelization*. Each task is assigned to one thread block and all *dimBlock *threads in the thread block cooperate to perform the task in parallel, exploiting the parallel characteristics of cells in the minor diagonals as shown in Additional file 1. In consideration of the varying cell numbers on different minor diagonals, each minor diagonal is considered to have a same *virtual cell number *that is equal to min(*l*_*a*_, *l*_*b*_) for two sequences of lengths *l*_*a *_and *l*_*b*_. Intermediate results vectors for the (*i*-2)^*th*^, (*i*-1)^*th *^and *i*^*th *^minor diagonals are allocated based on this virtual number. If all the real cells have been computed, it indicates the completion of the computing on the minor diagonal. After one minor diagonal is done, it swaps the intermediate results vectors, synchronizes all the threads in the thread block, and starts computing the next one.

Inter-task parallelization occupies more device memory but achieves better performance than intra-task parallelization. However, intra-task parallelization occupies significantly less device memory and therefore can support longer query/subject sequences. In our implementation, two stages are used: the first stage exploits inter-task parallelization and the second intra-task parallelization. A subject sequence length *threshold *is introduced to separate these two stages. For subject sequences of length less than or equal to *threshold*, the alignments with a query sequence are performed in the first stage in order to maximize the performance. The alignments of subject sequences of length greater than *threshold*, are carried out in the second stage. In our implementation, the threshold is set to 3,072, since up to 404,955 sequences (more than 99.86%) in Swiss-Prot release 56.6 (released on Dec. 16, 2008, comprising 146,166,984 amino acids in 405,506 sequences and having the longest sequence of 35,213 amino acids) are of length less than or equal to 3,072. In order to achieve high efficiency for inter-task parallelization, the runtime of all threads in a thread block should be roughly identical. We therefore order the subject sequences based on their lengths, while reading in the database. Hence, for two adjacent threads in a thread block, the difference value between the products of the lengths of the associated sequences is minimized. SW-CUDA [14] is based on the inter-task parallelization approach and the OpenGL GPU implementation presented in [13] uses the intra-task parallelization approach. Compared to [13] and [14], our implementation uses three techniques to improve performance: (i) coalesced subject sequence arrangement, (ii) coalesced global memory access and (iii) cell block division method, which are explained in detail in the following.

#### Coalesced subject sequence arrangement

For inter-task parallelization, sorted subject sequences are arranged in an array like a multi-layer bookcase (see Additional file 4(a)), where all symbols of a sequence are restricted to be stored in the same column from top to bottom and all sequences are arranged in increasing length order from left to right and top to bottom in the array. Sorted subject sequences for the intra-task parallelization are sequentially stored in an array row by row from the top-left corner to the bottom-right corner (see Additional file 4(b)), where all symbols of a sequence are restricted to be stored in the same row from left to right. Using these arrangement patterns for both parallelization methods, access to the subject sequences is coalesced for all threads in a half-warp, even if texture cache is not used. It is however important to utilize the texture cache, if the range limitation of texture reference allows, so as to achieve maximum performance on coalesced access patterns. A hash table records the location coordinate in the array and the length of each sequence, providing fast access to any sequence.

#### Coalesced global memory access

During the execution of the SW algorithm, additional memory is required to store intermediate alignment data. The size of this memory is *O*(min{*l*_*a*_, *l*_b_}) for two sequences of length *l*_*a *_and *l*_*b*_. To support much longer sequences, the global memory is used to store the intermediate results. To gain maximum bandwidth and best performance, all threads in a half-warp should access the intermediate results in global memory in a coalesced pattern. A prerequisite for coalescing is that the words accessed by all threads in a half-warp must lie in the same segment. The memory spaces referred to by the same variable names (not referring to same addresses) for all threads in a half-warp have to be allocated in the form of an array to keep them contiguous in address. Additional file 5 presents two global memory allocation patterns of a basic type vector variable of size *N *for *M *processing entities (threads or thread blocks, here). Inter-task parallelization exploits the pattern shown in Additional file 5(a), where a memory slot is allocated to a thread in a thread block and is indexed top-to-bottom, and the access to *MemSlot *using the same index for all threads in a half-warp is coalesced into one or two memory transactions depending on the compute capacity of devices. Intra-task parallelization exploits the pattern shown in Additional file 5(b), where a memory slot is allocated to a thread block and is indexed left-to-right, and the coalesced access is able to be obtained using the common global memory access pattern, i.e. that successive threads access the successive addresses in a memory slot. These two approaches work in a multi-pass fashion, where in every pass, a grid consisting of thread blocks whose number is equal to or less than the number of SMs are bound to the kernel and launched, and the memory allocated for one pass is multiplexed by the successive following passes, reducing the requirements for global memory.

#### Cell block division method

To maximize performance and to reduce the bandwidth demand of global memory, we propose a cell block division method for the inter-task parallelization, where the alignment matrix is divided into cell blocks of equal size. A cell block is a square matrix of size *n *× *n*. Assume that the lengths of a query sequence and a subject sequence are *qlen *and *slen*, respectively. In this case, both *qlen *and *slen *must be multiples of *n*. If the length is not a multiple of *n*, the sequence is padded with an appropriate number of dummy symbols. In order to keep the similarity score unchanged, the dummy symbol must be added to the scoring matrix and the score between the dummy symbol and itself or a real symbol is set to zero. For simplicity, assume that *qlen *and *slen *are multiples of *n*. Without cell block division, the computation of one cell, including the computation of the corresponding values in the *H*, *E *and *F *matrices, results in one load operation and one store operation for the intermediate results stored in the global memory. We define the runtime of one load operation to be *T*_*l*_, the runtime of one store operation to be *T*_*s *_and the computation time of one cell value to be *T*_*c*_. Then, without cell block division, the total runtime can be denoted as:

(2)

However, when using the cell block division method, the computation of *n *cells in one column (or row) in a cell block only requires one load operation and one store operation on the global memory instead of *n *load operations and *n *store operations. In this case, the total runtime can be denoted as:

(3)

Since one global memory access takes hundreds of clock cycles, the cell block division method leads to a signification reduction of the total runtime due to a reduction in the global memory accesses. However, the size of cell block is limited by the number of registers (or the amount of shared memory) available per thread. Therefore, this leads to the optimal cell block size of 8 × 8 for our implementation.

**Figure 1:**
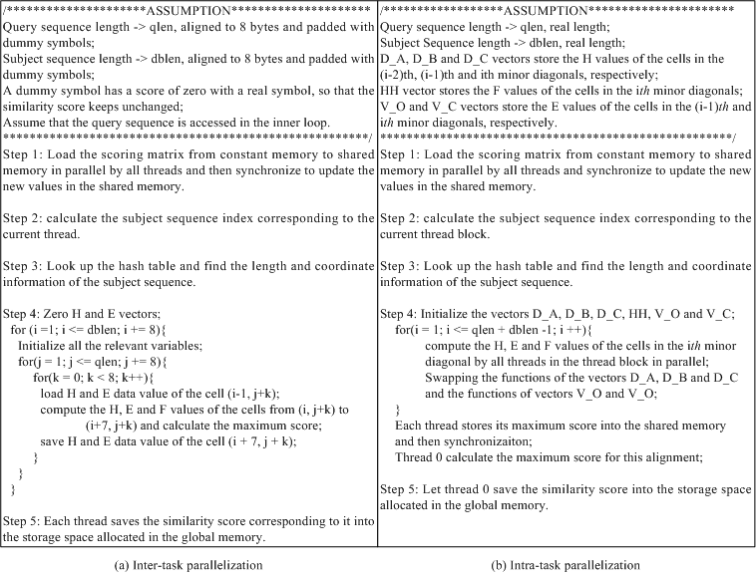
**Pseudocodes of the CUDA kernels for the inter-task and intra-task parallelization**.

Constant memory is exploited to store the gap penalties, scoring matrix and the query sequence. Before searching for a query sequence against the database, the query sequence is loaded into constant memory. The 64 KB memory capability of the constant memory makes it possible to accommodate much longer sequences. In our implementation, sequences of length up to 59K (see Table 1) can be supported. As mentioned above, as long as all threads in a half-warp read the same address in constant memory, the access is as fast as reading from registers. Placing the query sequence in constant memory provides a significant performance improvement as all threads in a warp on the common execution path read the same query sequence address. The scoring matrix is loaded into shared memory, as the performance of constant memory degrades linearly if multiple addresses are requested by threads. This is because threads may frequently access different addresses in the scoring matrix. The integer functions *max(x, y) *and *min(x, y) *in the CUDA runtime library are used to map them to a single instruction on the device. Figure 1 presents the pseudocodes of the CUDA kernels for the inter-task and intra-task parallelization.

**Table 1:** Comparison of supported maximum query sequence length

**Smith-Waterman Software**	**Maximum Sequence Length**
NCBI-BLAST [5]	Unlimited
SWPS3 [10]	10K
CBESW [12]	852
SW-CUDA [14]	2050
CUDASW++	59K

We also examined the SWAT-like optimizations [19] which resulted in worse performance. SWAT optimization is based on the following observation: for most cells in the matrix, *F *remains zero and does not contribute to the value of *H. F *only starts to influence the value of *H*, and needs to be recalculated sequentially, when *H *is greater than the threshold. However, due to the divergence of the execution paths of all threads in a warp when processing to decide whether or not it is required to recalculate the value of *F*, this approach leads to a performance reduction on CUDA-enabled GPUs.

## Results and discussion

To remove the dependency on the query sequences and the databases used for the different tests, *cell updates per second *(CUPS) is a commonly used performance measure in bioinformatics. A CUPS represents the time for a complete computation of one cell in matrix *H*, including all memory operations and the corresponding computation of the values in the *E *and *F *matrices. Given a query sequence of size *Q *and a database of size *D*, the GCUPS (billion cell updates per second) value is calculated by:

(4)

where *|Q| *is the total number of symbols in the query sequence, *|D| *is the total number of symbols in the database and *t *is the runtime in second. In this paper, for the single-GPU version, the runtime *t *includes the transfer time of the query sequences from host to GPU, the calculation time of the SW algorithm, and the transfer-back time of the scores; and for the multi-GPU version, the runtime *t *includes the creating and destroying time of host threads, the transfer time of the database sequences and query sequences from host memory to GPU, the calculation time of the SW algorithm, and the transfer-back time of the scores.

The performance of CUDASW++ is benchmarked and analyzed by searching for 25 sequences of length from 144 to 5,478 against Swiss-Prot release 56.6. The tests of the single-GPU version are carried out on a GTX 280 graphics card, with 30 SMs comprising 240 SPs and 1 GB RAM, installed in a PC with an AMD Opteron 248 2.2 GHz processor running the Linux OS. This graphics card has a core frequency of 602 MHz, a unified processors frequency of 1,296 MHz and a memory clock of 1,107 MHz. The tests of the multi-GPU version are carried out on a GTX 295 graphics card with two G80 GPU-chips on a single card, which consists of 480 SPs (240 SPs per GPU) and 1.8 GB RAM and is also installed in the above PC. This graphics card has a slightly lower clock frequencies compared to the GTX 280 (core frequency of 576 MHz, unified processors frequency of 1,242 MHz memory clock of 999 MHz). Maximal performance is achieved for a thread block size of 256 threads and a grid size equal to the number of SMs for both the single-GPU and multi-GPU versions. The scoring matrix BLOSUM45 is used with a gap penalty of 10-2 k. For the single-GPU version, it achieves a relatively constant performance for all 25 query sequences (see Table 2): with a highest performance of 9.660 GCUPS, a lowest performance of 9.039 GCUPS and an average performance of 9.509 GCUPS. For the multi-GPU version, the performance increases as the lengths of query sequences become longer, due to the overhead incurred mainly by the database loading from host memory to GPU and the host threads scheduling. It achieves a highest performance of 16.087 GCUPS, a lowest performance of 10.660 GCUPS and an average performance of 14.484 GCUPS.

**Table 2:** Performance Evaluation of CUDASW++

**Queries**	**Length**	**Single-GPU version**		**Multi-GPU version**	
		
		**Time(s)**	**GCUPS**	**Time(s)**	**GCUPS**
P02232	144	2.33	9.039	1.97	10.660
P01111	189	3.01	9.163	2.47	11.194
P05013	189	3.01	9.163	2.40	11.513
P14942	222	3.48	9.333	2.87	11.304
P00762	246	3.82	9.402	3.10	11.591
P07327	375	5.80	9.453	4.21	13.034
P01008	464	7.08	9.582	4.67	14.532
P10635	497	7.66	9.483	4.99	14.566
P25705	553	8.61	9.390	5.49	14.711
P03435	567	8.72	9.507	5.52	15.022
P42357	657	10.11	9.496	6.50	14.777
P21177	729	11.16	9.552	7.00	15.225
Q38941	850	13.02	9.539	8.15	15.235
O60341	852	13.03	9.556	8.11	15.355
P27895	1000	15.13	9.660	9.34	15.644
P07756	1500	22.74	9.642	14.02	15.640
P04775	2005	30.37	9.649	18.48	15.855
P19096	2504	37.89	9.659	22.79	16.058
P28167	3005	45.54	9.644	27.41	16.027
P0C6B8	3564	54.01	9.644	32.45	16.055
P20930	4061	61.60	9.635	36.94	16.070
P08519	4548	68.98	9.637	41.45	16.039
Q7TMA5	4743	71.91	9.640	43.18	16.054
P33450	5147	78.13	9.629	46.83	16.066
Q9UKN1	5478	83.15	9.629	49.77	16.087

**Figure 2:**
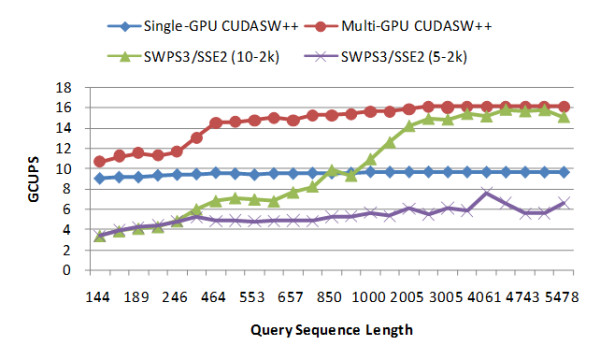
**Performance comparison between CUDASW++ and SWPS3 for x86/SSE2**.

**Figure 3:**
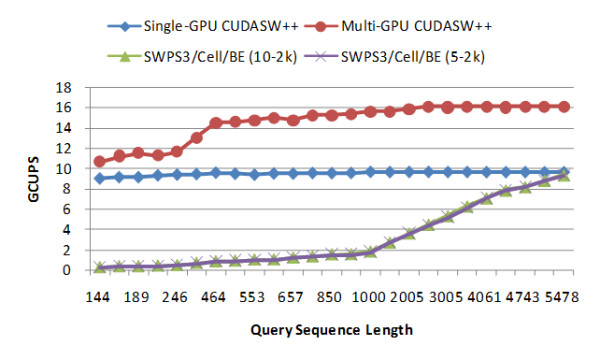
**Performance comparison between CUDASW++ and SWPS3 for Cell/BE**.

We next compare the performance of CUDASW++ with other publicly available implementations for protein database searches: SWPS3, SW-CUDA and NCBI-BLAST (version 2.2.19). All the following tests are performed against Swiss-Prot release 56.6. SWPS3 outperforms the other two publicly available Cell/BE implementations (Farrar [11] and CBESW [12]) and therefore we have decided not to include comparisons to [11] and [12]. SWPS3 for x86/SSE2 is tested on a Linux workstation with two Intel Xeon 3.0 GHz dual-core processors by running four threads and SWPS3 for Cell/BE is tested on a stand-alone PS3. The scoring matrix BLOSUM50 is used for the tests with a gap penalty of 10-2 k and 5-2 k respectively. All the other parameters are used by default. Figures 2 and 3 present the performance comparison between CUDASW++ and SWPS3 for x86/SSE2 and Cell/BE, respectively. From the figures, the performance of SWPS3 for x86/SSE2 is sensitive with respect to gap penalties, whereas, surprisingly, the gap penalties seem to have little impact on the performance of SWPS3 for Cell/BE. SWPS3 achieves a peak performance of up to 15 GCUPS for x86/SSE2 and a peak performance of up to 9 GCUPS for Cell/BE. The multi-GPU version outperforms SWPS3 for x86/SSE2 and Cell/BE for all the query sequences, but the single-GPU version only outperforms SWPS3 for Cell/BE. On average, compared to SWPS3 for x86/SSE2, the multi-GPU version runs about 1.46 times faster than SWPS3 with a gap penalty of 10-2 k and about 2.74 times faster than with a gap penalty of 5-2 k; compared to SWPS3 for Cell/BE, the multi-GPU version runs about 4.70 times faster than SWPS3 with a gap penalty of 10-2 k and about 4.73 times faster than SWPS3 with a gap penalty of 5-2 k.

**Figure 4:**
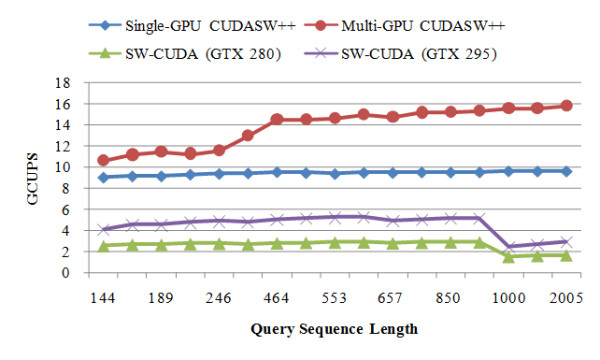
**Performance comparison between CUDASW++ and SW-CUDA**.

The performance of SW-CUDA is re-benchmarked using the scoring matrix BLOSUM50 with a gap penalty of 10-2 k on the GTX 280 and GTX 295 graphics cards, respectively, installed in the above PC with an AMD processor. The performance comparison between CUDASW++ and SW-CUDA is shown in Figure 4. As can be seen from the figure, even the single-GPU version running on the GTX 280 outperforms SW-CUDA running on the GTX 295 for all the query sequences supported by the latter. Compared to SW-CUDA running on the GTX 280 and on the GTX 295, respectively, the single-GPU version runs about 3.65 and 2.08 times faster on average and up to 6.34 and 3.85 times faster for longer sequences, and the multi-GPU version runs about 5.36 and 3.05 times faster on average and up to 10.27 and 6.24 times faster for longer sequences.

**Figure 5:**
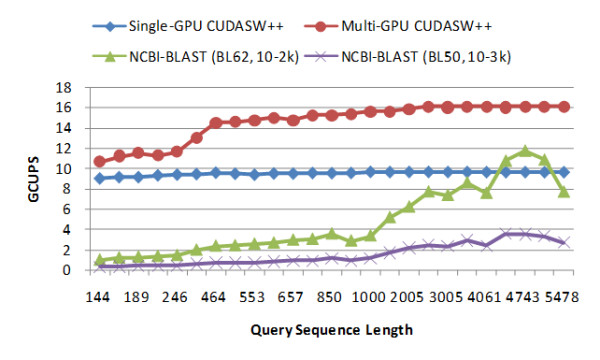
**Performance comparison between CUDASW++ and NCBI-BLAST**.

The performance of NCBI-BLAST is benchmarked on a Linux workstation with an Intel Xeon 3.0 GHz dual-core processor. The scoring matrices BLOSUM62 with a gap penalty of 10-2 k and BLOSUM50 with a gap penalty of 10-3 k are used for the tests. All the other parameters are used by default. Figure 5 presents the performance comparison between CUDASW++ and NCBI-BLAST. The NCBI-BLAST seems to be highly sensitive in terms of scoring matrix and gap penalties. As observed in our tests, it runs about 3 times faster using BLOSUM62 than using BLOSUM50 on average. NCBI-BLAST shows increasing performance as the query sequence lengths increase due to more effective filtration. Compared to NCBI-BLAST using BLOSUM50, the two versions of CUDASW++ outperform it for all the query sequences of length from 144 to 5,478; Compared to NCBI-BLAST using BLOSUM62, the multi-GPU version outperforms it for all the query sequences and for the sequences of length less than or equal to 4,061, the single-GPU version achieves better performance but for very long sequences of length greater than 4,061, NCBI-BLAST outperforms it. Considering that there are only 258 sequences of length greater than 4,061 in Swiss-Prot release 56.6 (the percentage is about 0.063%), the overall performance of the single-GPU version is significantly better. On average, the single-GPU version runs about 6.27 times faster than NCBI-BLAST using BLOSUM50 and about 2.02 times faster than using BLOSUM62; and the multi-GPU version runs about 9.55 times faster than NCBI-BLAST using BLOSUM50 and about 3.08 times faster than using BLOSUM62.

## Conclusion

In this paper, we have demonstrated the compute capability of CUDA-enabled GPUs for accelerating SW sequence database searches. CUDASW++ is targeted for CUDA-enabled GPUs with compute capability 1.2 and higher and supports query sequences of length up to 59K, far longer than the maximum sequence length 35,213 in Swiss-Prot release 56.6. Two versions of CUDASW++ are implemented and benchmarked: a single-GPU version and a multi-GPU version. For the single-GPU version, it achieves consistent performance for query sequences of length varying from 144 to 5,478, where the performance figures vary from a low of 9.039 GCUPS to a high of 9.660 GCUPS, with an average performance of 9.509 GCUPS; for the multi-GPU version, it achieves an increasing performance as the lengths of the query sequences increase from 144 to 5,478, where the performance figures vary from a low of 10.660 GCUPS to a high of 16.087 GCUPS, with an average performance of 10.660 GCUPS. CUDASW++ outperforms previous SW sequence database search implementations on GPUs and other implementations using SSE2, Cell/B.E or heuristics.

Due to the rapid growth in biological sequence databases, even more powerful high-performance solutions will be demanded in the near future. Since computer architectures are rapidly developing towards many-core systems, future solutions are likely to be aimed at exploiting the compute capability of these high-performance architectures. Our results on GPU show that it is possible to improve the performance of biological algorithms by making full use of the compute characteristics of the underlying commodity hardware and further, our results are especially encouraging since GPU performance grows faster than multi-core CPUs [20].

## Availability and requirements

• **Project name**: CUDASW++

• **Project home page**: 

• **Operating System**: Linux

• **Programming language**: CUDA and C

• **Other requirements**: CUDA SDK and Toolkits 2.0 or higher

• **License**: none

## Abbreviations

CPU: Central Processing Unit; CUDA: Compute Unified Device Architecture; Cell/BE: Cell Broadband Engine Architecture; FPGA: Field-Programmable Gate Array; GCPUS: Billion Cell Updates per Second; GPL: General Public License; GPU: Graphics Processing Unit; GTX 280: NVIDIA GeForce GTX 280; GTX 295: NVIDIA GeForce GTX 295; MPI: Message Passing Interface; OpenGL: Open Graphics Library; OS: Operating System; PBSM: Per-block Shared Memory; PS3: PlayStation 3; RAM: Random Access Memory; SIMD: Single Instruction Multiple Data; SIMT: Single-instruction, Multiple-thread; SM: Streaming Multiprocessor; SP: Scalar Processor; SSE2: Streaming SIMD Extensions 2; SW: Smith-Waterman.

## Competing interests

The authors declare that they have no competing interests.

## Authors' contributions

YL conceptualized the study, carried out the design and implementation of the algorithm, performed benchmark tests, analyzed the results and drafted the manuscript; DLM conceptualized the study and contributed to the revising of the manuscript; BS conceptualized the study, participated in the algorithm optimization and analysis of the results and contributed to the revising of the manuscript. All authors read and approved the final manuscript.

## Supplementary Material

Additional file 1 **Data dependencies in the alignment matrix for SW algorithm**. This figure demonstrates the data dependencies in the alignment matrix for the Smith-Waterman algorithm. Click here for file

Additional file 2 **Execution model of CUDA-enabled GPUs**. This figure demonstrates the execution model of CUDA-enabled GPUs, where serial code executes on the host while parallel code executes on the device. Click here for file

Additional file 3 **Hardware model (Tesla) of CUDA-enabled GPUs**. This figure demonstrates the hardware model (Tesla) of CUDA-enabled GPUs consisting of a set of SIMT multiprocessors with on-chip shared memory, constant cache, texture cache and device memory. Click here for file

Additional file 4 **The arrangement of the subject sequences in the database**. This figure demonstrates the arrangement of the subject sequences in the database for the inter-task and intra-task parallelization: (a) subject sequences arrangement for the inter-task parallelization and (b) subject sequences arrangement for the intra-task parallelization. Click here for file

Additional file 5 **Two global memory allocation patterns for processing entities**. This figure demonstrates two global memory allocation patterns of a basic type variable of size *N *for *M *processing entities (threads or thread blocks). Click here for file
