# Supplementary figures and images for: CUDASW++: optimizing Smith-Waterman sequence database searches for CUDA-enabled graphics processing units
Source: BMC Res Notes. 2009 May 6;2:73. doi: 10.1186/1756-0500-2-73 (PMC2694204; doi:10.1186/1756-0500-2-73)

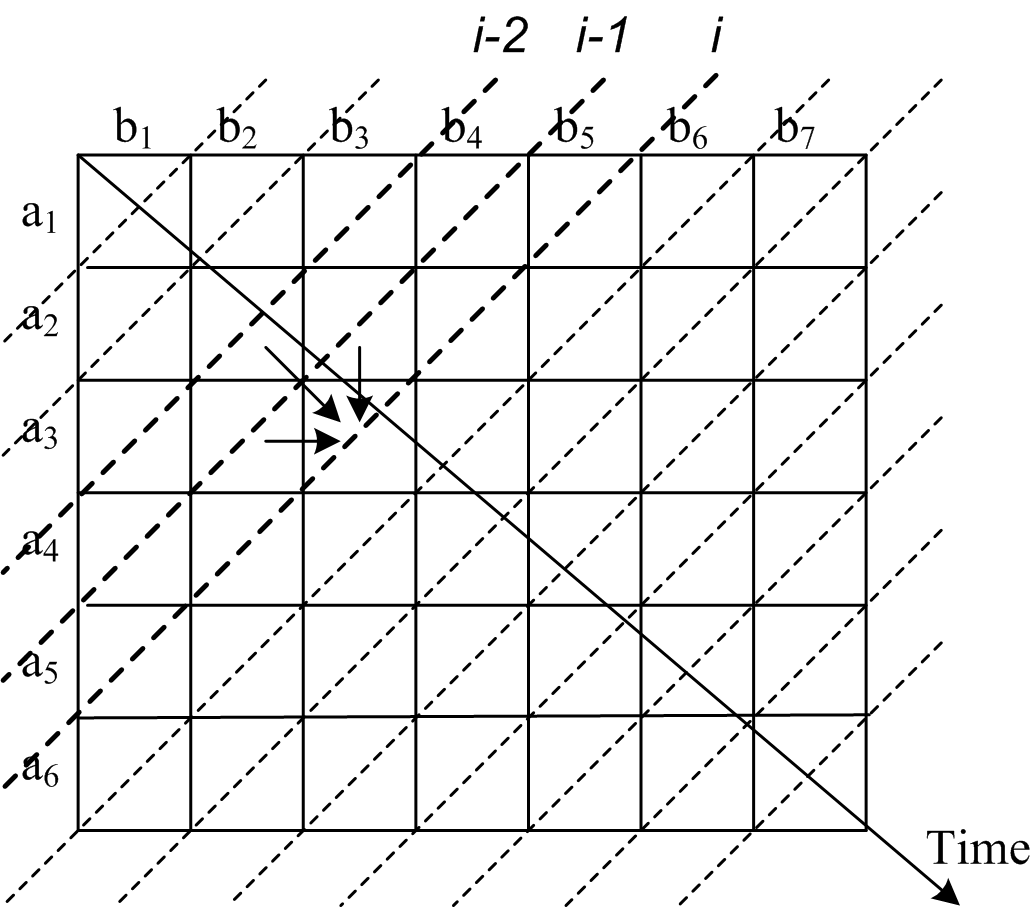

Supplement: Additional file 1 — Data dependencies in the alignment matrix for SW algorithm. This figure demonstrates the data dependencies in the alignment matrix for the Smith-Waterman algorithm. [file 1756-0500-2-73-S1.png]

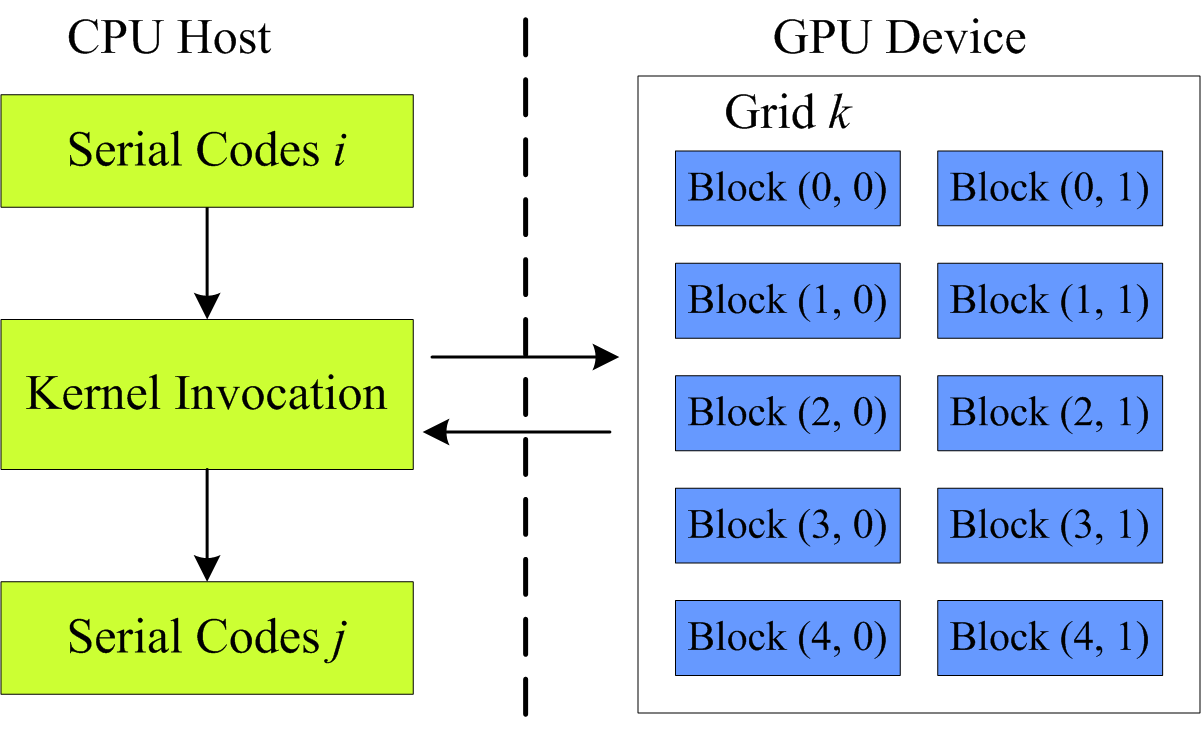

Supplement: Additional file 2 — Execution model of CUDA-enabled GPUs. This figure demonstrates the execution model of CUDA-enabled GPUs, where serial code executes on the host while parallel code executes on the device. [file 1756-0500-2-73-S2.png]

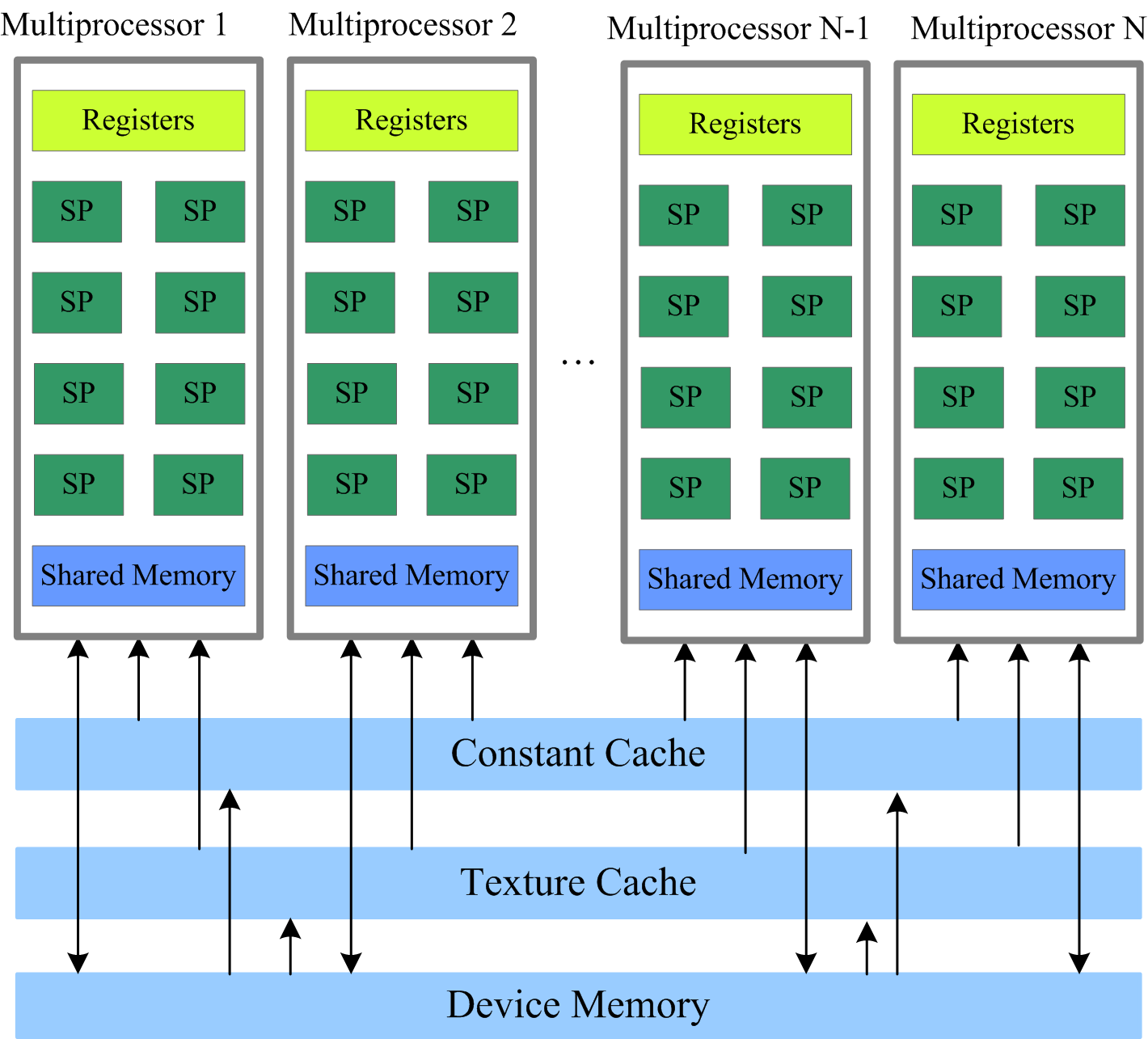

Supplement: Additional file 3 — Hardware model (Tesla) of CUDA-enabled GPUs. This figure demonstrates the hardware model (Tesla) of CUDA-enabled GPUs consisting of a set of SIMT multiprocessors with on-chip shared memory, constant cache, texture cache and device memory. [file 1756-0500-2-73-S3.png]

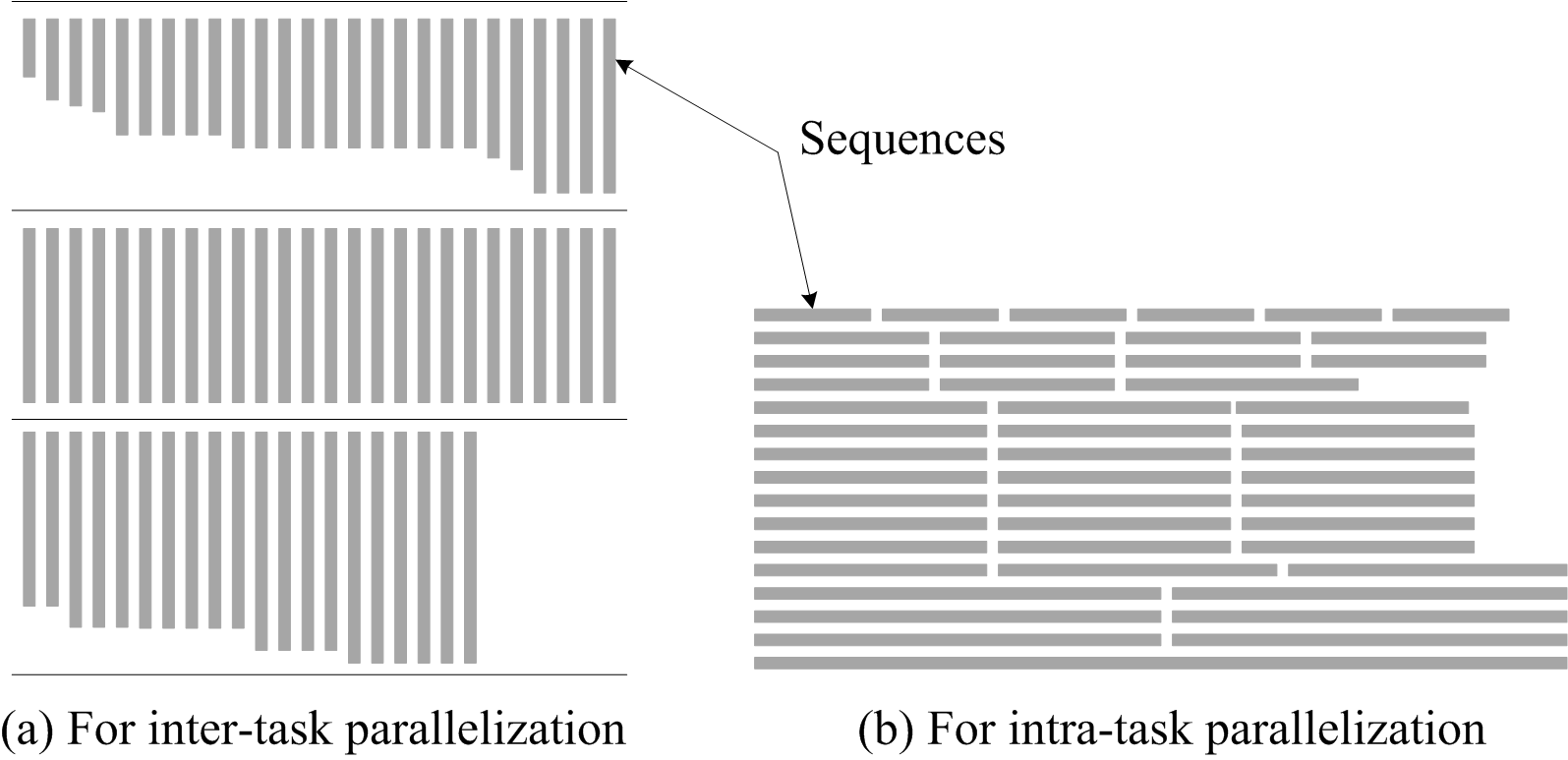

Supplement: Additional file 4 — The arrangement of the subject sequences in the database. This figure demonstrates the arrangement of the subject sequences in the database for the inter-task and intra-task parallelization: (a) subject sequences arrangement for the inter-task parallelization and (b) subject sequences arrangement for the intra-task parallelization. [file 1756-0500-2-73-S4.png]

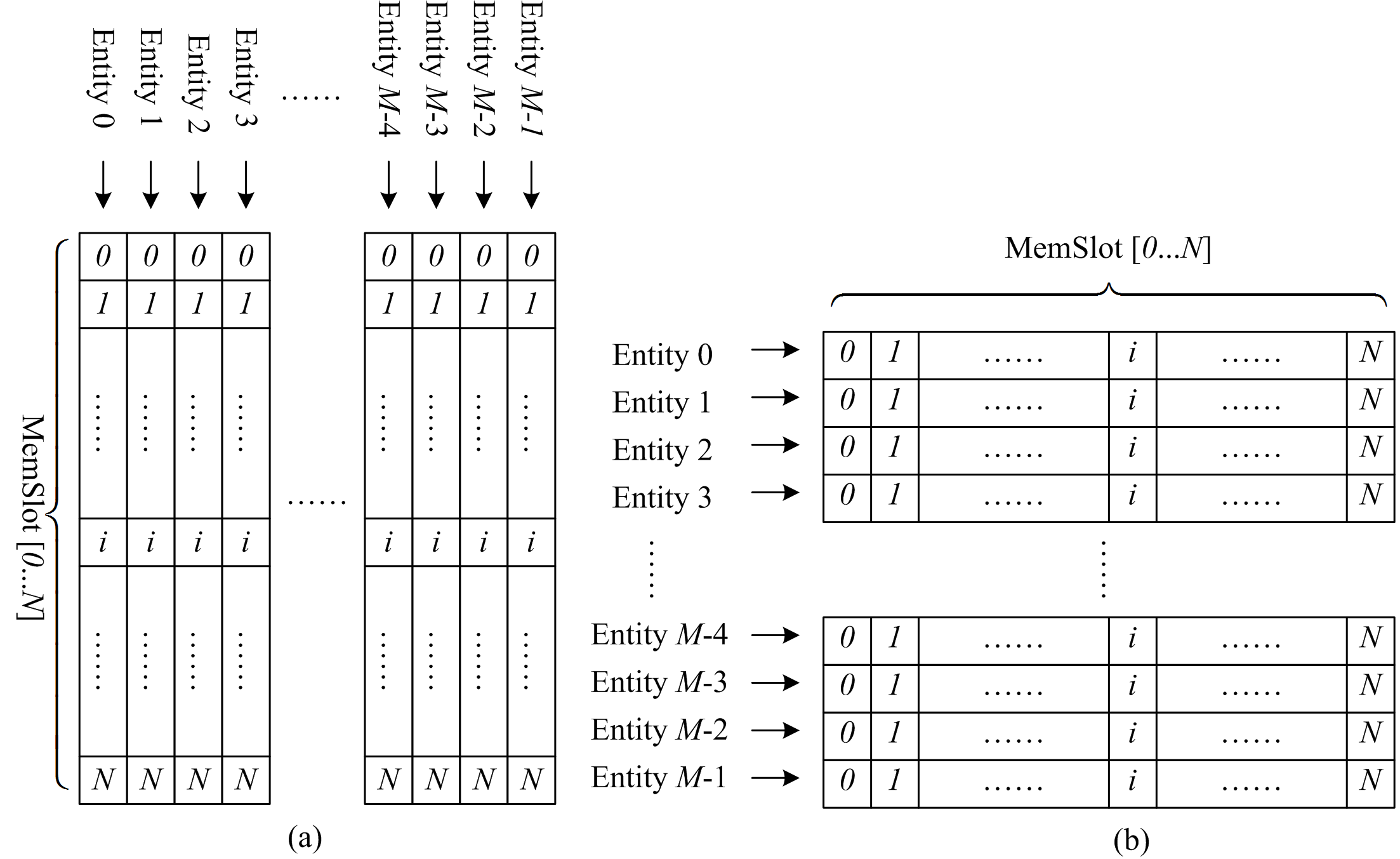

Supplement: Additional file 5 — Two global memory allocation patterns for processing entities. This figure demonstrates two global memory allocation patterns of a basic type variable of size N for M processing entities (threads or thread blocks). [file 1756-0500-2-73-S5.png]
